# Fatal Mixed *Plasmodium* Infection in Traveler Returning to Colombia from Comoros Islands, 2024

**DOI:** 10.3201/eid3101.241491

**Published:** 2025-01

**Authors:** Leidy J. Medina-Lozano, Sergio Andrés Bolívar Lozano, Carolina Guavita, Milena Camargo, Luz Helena Patiño, Juan David Ramírez, Diana Carolina Gutiérrez-González, Álvaro A. Faccini-Martínez

**Affiliations:** Hospital Militar Central, Bogotá, Colombia (L.J. Medina-Lozano, S.A. Bolívar Lozano, C. Guavita, D.C. Gutiérrez-González, Á.A. Faccini-Martínez); Universidad Militar Nueva Granada, Bogotá (S.A. Bolívar Lozano, C. Guavita, Á.A. Faccini-Martínez); Universidad del Rosario, Bogotá (M. Camargo, L.H. Patiño, J.D. Ramírez); Icahn School of Medicine at Mount Sinai, New York, New York, USA (J.D. Ramírez)

**Keywords:** Malaria, Plasmodium vivax, Plasmodium falciparum, travel-related illness, parasites, Comoros, Colombia

## Abstract

During 2014–2022, only *Plasmodium falciparum* malaria cases were reported in the Comoro Islands. We report a fatal case of mixed *Plasmodium* malaria infection in a traveler returning from the Comoros to Colombia in 2024, highlighting the need to strengthen laboratory detection and identification of *Plasmodium* spp. in sub-Saharan Africa.

Malaria is the most common life-threatening tropical disease associated with fever among returned travelers from sub-Saharan Africa. During 2010–2013, according to the World Malaria Report 2023, the Comoros Islands reported a total of 144,546 cases of *Plasmodium falciparum* infection and 1,571 cases of *P. vivax* infection (*1*). Nevertheless, during 2014–2022, only *P. falciparum* cases were reported, without *P. vivax* cases or mixed infections (*1*).

Data collected by the GeoSentinel Surveillance Network for 1,415 ill travelers returning from Indian Ocean islands during 1997–2010 indicated that the proportion of mosquitoborne infections (including malaria) was higher among travelers to the Comoros than among other travelers (*2*). At the same time, studies published in the past 10 years reported malaria cases exported from the Comoros to other countries during 1999–2021, mainly to territories of France (France, Réunion, and Mayotte) and 1 case to Japan; the most common etiologic agent was *P. falciparum* (≈255 cases), followed by *P. ovale* (≈19 cases) and *P. vivax* (≈11 cases) (*3*–*7*). We report a case of fatal mixed *Plasmodium* malaria infection in a man who returned to Colombia from the Comoros in 2024.

On June 14, 2024, an otherwise healthy 50-year-old male former military service member sought care at a primary care center in Bogotá (capital city of Colombia) after 7 days of fever (up to 39°C), chills, diaphoresis, myalgias, arthralgias, and headache. He reported a 2-day history of epigastric pain, loose stools, and dark urine. His illness was considered an unspecific viral infection, and he was discharged. His signs/symptoms had begun 10 days after he returned from Grande Comore Island, where he had stayed for 2 weeks while providing military training. Until his travel to the Comoros, he had not been in another *P. vivax*/*P. falciparum*–endemic area in the previous 5 years. On June 15, 2024, he was admitted to Hospital Militar Central, a reference military hospital in Bogotá, for a syncopal episode, disorientation, and jaundice. Physical examination revealed hypothermia, tachycardia with Kussmaul breathing, and reduced oxygen saturation. The patient was jaundiced and stuporous with no bleeding.

Laboratory tests revealed leukocytosis, anemia, severe thrombocytopenia, malarial hepatopathy, renal impairment, metabolic acidosis, and hyperlactatemia (Table). Thick and thin blood smears showed *P. falciparum* (17,840 trophozoites/μL; parasitemia of 0.35%) with gametocytes and *P. vivax* (8,320 trophozoites/μL). Severe malaria was diagnosed, and treatment with intravenous artesunate was initiated (2.4 mg/kg) in addition to fluid resuscitation and invasive mechanical ventilation support. However, the patient experienced 2 episodes of cardiopulmonary arrest and died. Autopsy and histopathologic examination of heart and brain samples revealed multiple parasitic structures compatible with *Plasmodium* trophozoites (Appendix Figures 1, 2). PCR performed on blood smears confirmed the presence of *P. falciparum* and *P. vivax* (Appendix). DNA gene fragments from the small subunit rRNA 18S gene were sequenced from the positive specimens, and phylogenetic analyses positioned the obtained sequences in the same subclade as *P. falciparum* sequences detected in South Africa and as *P. vivax* sequences detected in Cameroon, Nigeria, China, and India (Figure; Appendix). Sequences were deposited in GenBank (*P. falciparum* accession no. PQ408861, *P. vivax* accession no. PQ408862).

**Table:** Laboratory parameters of man with mixed *Plasmodium* malaria who had returned to Colombia from the Comoro Islands, June 15, 2024

Parameter	Value (reference range)
Leukocytes, ×10^9^ cells/L	28.3 (4.5–11.0)
Neutrophils, ×10^9^ cells/L	19.2 (2.0–8.0)
Lymphocytes, ×10^9^ cells/L	5.68 (0.9–4.5)
Hemoglobin, g/dl	8.3 (12.1–16.6)
Platelets, ×10^9^/L	12 (150–450)
Aspartate aminotransferase, U/L	109 (0–40)
Alanine aminotransferase, U/L	75 (0–41)
Total bilirubin, mg/dL	8.9 (0.01–1.1)
Conjugated bilirubin, mg/dL	7.0 (0.25–0.3)
Unconjugated bilirubin, mg/dL	1.9 (0.25–0.8)
Lactate dehydrogenase, U/L	918 (5–248)
Urobilinogen, mg/dL	8 (0.1–1.8)
Creatinine, mg/dL	2.92 (0.6–1.1)
Urea nitrogen, mg/dL	97 (8–23)
C-reactive protein, mg/dL	19.6 (0–0.5)
pH	7.03 (7.35–7.45)
Arterial partial pressure of carbon dioxide, mm Hg	13 (29–31)
Bicarbonate, mmol/L	3.4 (19–21)
Lactate, mmol/L	17 (0.36–0.75)

**Figure:**
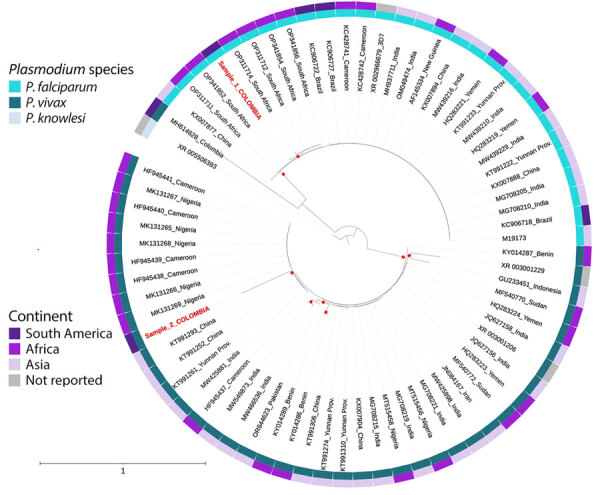
Phylogenetic tree of the DNA sequences of *Plasmodium falciparum* and *Plasmodium vivax* (red text) isolated from traveler returning to Colombia from the Comoros Islands, 2024, and compared with sequences reported from other countries. The red points on the tree represent bootstraps >80%.

In the most recent study that used PCR to assess the distribution of *Plasmodium* spp. on Grande Comore Island, among 159 positive samples collected during 2012–2013, nearly all (98.11%) were positive for *P. falciparum* and only 1.25% were positive for *P. vivax* (*8*). At that time, the authors indicated that routinely, without PCR testing, the rapid diagnostic tests used in the Comoros were able to identify *P. falciparum* but no other *Plasmodium* spp. (*8*), which is in accordance with a recent published editorial that discusses the contemporary concern with regard to the need to re-evaluate the spread of *P. vivax* in sub-Saharan Africa (*9*). The editorial mentioned that during 2017–2021, among 1.57 billion malaria rapid diagnostic tests purchased for use by sub-Saharan Africa national malaria control programs, 79.4% were focused on identifying *P. falciparum* and the remainder were combination tests lacking *P. vivax* specificity; thus, the predominant approach for malaria diagnosis across Africa was unable to specifically detect *P. vivax* (*9*). Our report highlights the value of strengthening laboratory diagnostic tools with good performance for detecting and accurately identifying *Plasmodium* spp. in clinical settings and of conducting more genetic-epidemiologic studies in the Comoros and other sub-Saharan Africa countries.

Appendix Additional information for report of fatal mixed *Plasmodium* infection in traveler returning to Colombia from the Comoros Islands, 2024.

## References

[1] World Health Organization. World malaria report [cited 2024 Oct 1]. https://www.who.int/teams/global-malaria-programme/reports/world-malaria-report-2023

[2] Savini H, Gautret P, Gaudart J, Field V, Castelli F, López-Vélez R, et al.; GeoSentinel Surveillance Network. Travel-associated diseases, Indian Ocean Islands, 1997-2010. Emerg Infect Dis. 2013;19:1297–301. 10.3201/eid1908.121739 23876977 PMC3739505

[3] Parola P, Gazin P, Pradines B, Parzy D, Delmont J, Brouqui P. Marseilles: a surveillance site for malaria from the Comoros Islands. J Travel Med. 2004;11:184–6. 10.2310/7060.2004.18470 15710062

[4] Tsukadaira A, Sekiguchi T, Ashida T, Murashita C, Itoh N, Kobayashi M, et al. A pregnant Japanese woman returning from Africa with recurrent fevers. Int Med Case Rep J. 2011;4:83–5. 10.2147/IMCRJ.S26997 23754912 PMC3658244

[5] Demaison X, Rapp C, de Laval F, Simon F. Malaria attacks due to *P. vivax* or *P. ovale* in two French military teaching hospitals (2000 to 2009). Med Mal Infect. 2013;43:152–8. 10.1016/j.medmal.2013.01.005 23561033

[6] Pagès F, Houze S, Kurtkowiak B, Balleydier E, Chieze F, Filleul L. Status of imported malaria on Réunion Island in 2016. Malar J. 2018;17:210. 10.1186/s12936-018-2345-y 29793505 PMC5968708

[7] Lepère JF, Collet L, Idaroussi AB, Pradines B. Mayotte, a malaria-free island at last [in French]. Med Trop Sante Int. 2023;3:mtsi.v3i1.2023.289. 10.48327/mtsi.v3i1.2023.289 PMC10300655 37389376

[8] Papa Mze N, Ahouidi AD, Diedhiou CK, Silai R, Diallo M, Ndiaye D, et al. Distribution of *Plasmodium* species on the island of Grande Comore on the basis of DNA extracted from rapid diagnostic tests. Parasite. 2016;23:34. 10.1051/parasite/2016034 27561250 PMC5000577

[9] Oboh-Imafidon MA, Zimmerman PA. *Plasmodium vivax* in sub-Saharan Africa: an advancing threat to malaria elimination? Am J Trop Med Hyg. 2023;109:497–8. 10.4269/ajtmh.23-0523 37640286 PMC10484284

