# Supplementary material for: Fatal Mixed Plasmodium Infection in Traveler Returning to Colombia from Comoros Islands, 2024
Source: Emerg Infect Dis. 2025 Jan;31(1):178–80. doi: 10.3201/eid3101.241491 (PMC11682824; doi:10.3201/eid3101.241491)
Supplement: Appendix — Additional information for report of fatal mixed Plasmodium infection in traveler returning to Colombia from the Comoros Islands, 2024. [file 24-1491-Techapp-s1.pdf]

Article DOI: https://doi.org/10.3201/eid3101.241491

EID cannot ensure accessibility for supplementary materials supplied by authors. Readers who have difficulty accessing supplementary content should contact the authors for assistance.

Fatal Mixed *Plasmodium* Infection in Traveler Returning to Colombia from Comoros Islands, 2024

Appendix

Isolation of parasite DNA and identification of *P. falciparum* and *P. vivax*

DNA was extracted from Wright-Giemsa-stained slides, which had been confirmed positive for *Plasmodium* sp. by microscopy. The extraction was performed using the High Pure PCR Template Preparation Kit (Roche) according to the manufacturer's instructions with minor modifications. Initially, the slides were immersed in xylene (Fisher Scientific) for 5 seconds, followed by rinsing with running water. Lysis buffer was then added for 5 minutes. The supernatant was collected and transferred into a 1.5-ml microtube. Subsequent steps followed the manufacturer's protocol.

P. falciparum and *P. vivax* were identified by nested PCR. The first round of PCR targeted the amplification of an 18S rRNA fragment for genus-level detection, while a second amplification (using the first PCR product as the template) was performed to identify the specific *Plasmodium* species using species-specific primers previously reported (Appendix Table 1) (*1*).

All PCRs were performed under the following conditions: 1X GoTaq® Green Master Mix (Promega), 1 µM of each primer, and 2 µL of template in a final volume of 12 µL. The thermocycling profile followed previously published protocols (*1*). All products obtained were analyzed in 2% agarose gels, stained with SYBR safe (Invitrogen). Finally, PCR products were purified using EXOSAP (Affymetrix) and sequenced in both directions with the BigDye Terminator kit (Macrogen, Seoul, South Korea).

Phylogenetic Analysis

The sequences obtained in this study were edited and assembled using UGENE software (*2*). For phylogenetic reconstructions, sequences of the 18S rRNA gene for *P. falciparum* and *P. vivax* were obtained from GenBank. These sequences were reported from African countries (including South Africa, Cameroon, and Nigeria), Asian countries (such as India, Yemen, China, and Pakistan), as well as from Colombia and Brazil in South America. Subsequently phylogenetic analyses were performed based on sequences aligned using CLUSTAL W (*3*). A maximum likelihood tree was constructed with the aligned sequences using the IQtree tool (*4*). The bootstrap method (BT, 1,000 replicates) was used to assess the robustness of the nodes, defining each cluster with a BT >80%. The phylogenetic trees were graphically visualized using the web-based tool Interactive Tree Of Life V3 (http://itol.embl.de) (*5*).

Identification of *P. falciparum* and *P. vivax* and phylogenetic analysis

For type-specific identification, the results showed amplification of *P. falciparum* and *P. vivax* in all extracted slides. Regarding Sanger sequencing, once the sequences were received, they were verified, and a consensus sequence was generated for each using the UGENE program (*2*). Subsequently, a BLAST search (https://blast.ncbi.nlm.nih.gov/Blast.cgi) was performed to identify the species present. The results showed 99.4% identity for *P. falciparum* and 100% identity for *P. vivax*.

The consensus sequence obtained from Sanger sequencing for each *Plasmodium* species from the analyzed sample was used to construct a phylogenetic tree. The tree included *18S ribosomal RNA* gene sequences from the amplified fragment, publicly available for *P. falciparum* (n = 29) and *P. vivax* (n = 42) (Appendix Table 2). The phylogenetic tree was constructed using IQtree with 1,000 bootstrap replicates. The tree topology revealed that the *P. falciparum* sample (identified in the tree as Sample_1_COLOMBIA) grouped with sequences from South Africa. For the *P. vivax* sequence (identified in the tree as Sample_2_COLOMBIA), the results showed it clustered with sequences from Cameroon, Nigeria, China and India. Red dots on the tree indicate bootstrap support of 80%. The newly generated 18S rRNA gene sequences of *P. falciparum* and *P. vivax* were deposited in GenBank (accession numbers: PQ408861 and PQ408862 respectively).

References

1. Kavunga-Membo H, Ilombe G, Masumu J, Matangila J, Imponge J, Manzambi E, et al. Molecular identification of Plasmodium species in symptomatic children of Democratic Republic of Congo. Malar J. 2018;17:334. PubMed https://doi.org/10.1186/s12936-018-2480-5

2. Okonechnikov K, Golosova O, Fursov M; UGENE team. Unipro UGENE: a unified bioinformatics toolkit. Bioinformatics. 2012;28:1166–7. PubMed https://doi.org/10.1093/bioinformatics/bts091

3. Thompson JD, Higgins DG, Gibson TJ. CLUSTAL W: improving the sensitivity of progressive multiple sequence alignment through sequence weighting, position-specific gap penalties and weight matrix choice. Nucleic Acids Res. 1994;22:4673–80. PubMed https://doi.org/10.1093/nar/22.22.4673

4. Nguyen LT, Schmidt HA, von Haeseler A, Minh BQ. IQ-TREE: a fast and effective stochastic algorithm for estimating maximum-likelihood phylogenies. Mol Biol Evol. 2015;32:268–74. PubMed https://doi.org/10.1093/molbev/msu300

5. Letunic I, Bork P. Interactive tree of life (iTOL) v3: an online tool for the display and annotation of phylogenetic and other trees. Nucleic Acids Res. 2016;44(W1):W242-5. PubMed https://doi.org/10.1093/nar/gkw290

Appendix Table 1. Primers used in this study

Plasmodium species	Primers	Sequence	Product size
P. falciparum	rFAL-F	CTTTTGAGAGGTTTTGTTACTTTGAGTAA	205 pb
	rFAL-R	TATTCCATGCTGTAGTATTCAAACAAAA	
P. vivax	rVIV-F	ACGCTTCTAGCTTAATCCACATAACT	120 pb
	rVIV-R	ATTTACTCAAAGTAACAAGGACTTCCAAGC	

Appendix Table 2. Accession number of the sequences used in phylogenetic reconstruction

GenBank number	Species	Country
XR 002966679.1	*P. falciparum*	Not reported
OP341856.1	*P. falciparum*	South Africa
OP341854.1	*P. falciparum*	South Africa
OP341852.1	*P. falciparum*	South Africa
OP311714.1	*P. falciparum*	South Africa
OP311712.1	*P. falciparum*	South Africa
OP311711.1	*P. falciparum*	South Africa
OM049474.1	*P. falciparum*	India
MW439229.1	*P. falciparum*	India
MW439216.1	*P. falciparum*	India
MH937711.1	*P. falciparum*	India
MG708205.1	*P. falciparum*	India
KX007894.1	*P. falciparum*	China
KX007888.1	*P. falciparum*	China
KT991222.1	*P. falciparum*	Yunnan Province
KC906727.1	*P. falciparum*	Brazil
KC906722.1	*P. falciparum*	Brazil
KC906718.1	*P. falciparum*	Brazil
KC428742.1	*P. falciparum*	Cameroon
KC428741.1	*P. falciparum*	Cameroon
HQ283221.1	*P. falciparum*	Yemen

GenBank number	Species	Country
HQ283219.1	*P. falciparum*	Yemen
AF145334.1	*P. falciparum*	New Guinea
MW439210.1	*P. falciparum*	India
MG708210.1	*P. falciparum*	India
M19173.1	*P. falciparum*	Not reported
KX007877.1	*P. falciparum*	China
KT991233.1	*P. falciparum*	Yunnan Province
XR 003001229.1	*P. vivax*	Not reported
XR 003001206.1	*P. vivax*	Not reported
OR644623.1	*P. vivax*	Pakistan
MW549873.1	*P. vivax*	India
MW466536.1	*P. vivax*	India
MW425898.1	*P. vivax*	India
MW425881.1	*P. vivax*	India
MT515458.1	*P. vivax*	Nigeria
MH614628.1	*P. vivax*	Colombia
MG708221.1	*P. vivax*	India
MG708219.1	*P. vivax*	India
MG708215.1	*P. vivax*	India
MF540772.1	*P. vivax*	Sudan
MF540770.1	*P. vivax*	Sudan
KY014289.1	*P. vivax*	Benin
KY014287.1	*P. vivax*	Benin
KX007904.1	*P. vivax*	China
KT991310.1	*P. vivax*	Yunnan Province
KT991306.1	*P. vivax*	China
KT991293.1	*P. vivax*	China
KT991274.1	*P. vivax*	Yunnan Province
KT991261.1	*P. vivax*	Yunnan Province
KT991252.1	*P. vivax*	China
JQ627158.1	*P. vivax*	India
JQ627156.1	*P. vivax*	India
JN084167.1	*P. vivax*	Iran
HQ283224.1	*P. vivax*	Yemen
HQ283223.1	*P. vivax*	Yemen
GU233451.1	*P. vivax*	Indonesia
MT515456.1	*P. vivax*	Nigeria
KY014286.1	*P. vivax*	Benin
HF945441.1	*P. vivax*	Cameroon
HF945437.1	*P. vivax*	Cameroon
HF945438.1	*P. vivax*	Cameroon
HF945439.1	*P. vivax*	Cameroon
HF945440.1	*P. vivax*	Cameroon
MK131265.1	*P. vivax*	Nigeria
MK131266.1	*P. vivax*	Nigeria
MK131267.1	*P. vivax*	Nigeria
MK131268.1	*P. vivax*	Nigeria
MK131269.1	*P. vivax*	Nigeria
XR 005506393.1	*P. knowlesi*	Not reported

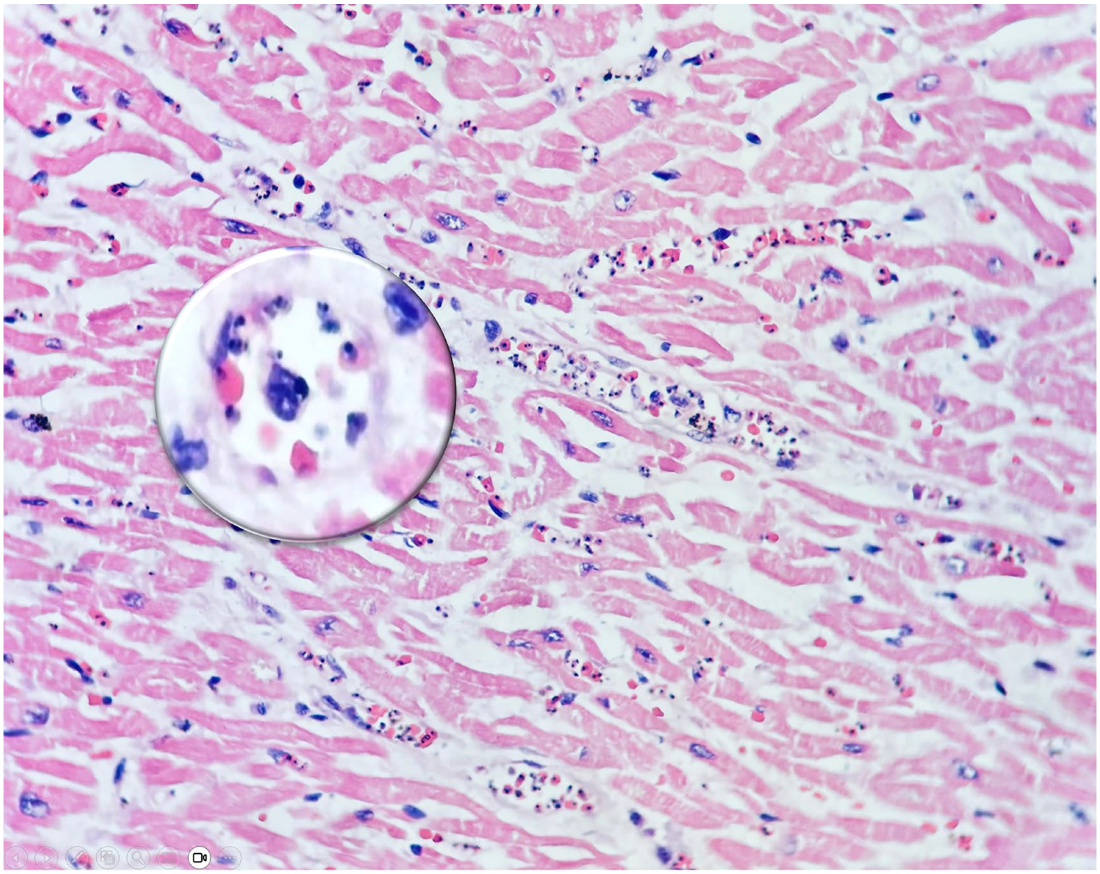

Appendix Figure 1. Photograph of myocardium at 400×, with Hematoxylin & Eosin staining, shows hypertrophic cardiomyocytes; abundant parasitic structures compatible with trophozoites are recognized in capillary lumens. The magnification image highlights the presence of intracellular parasites.

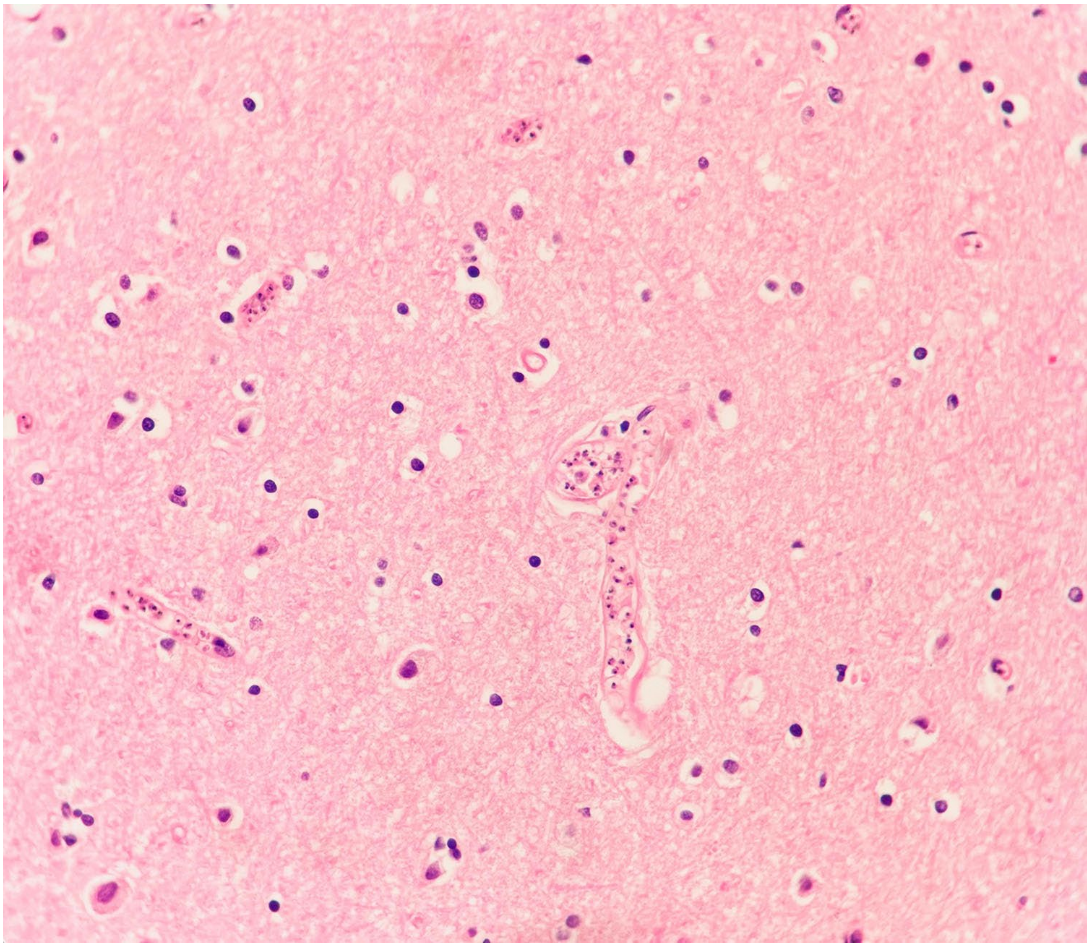

Appendix Figure 2. Photograph of brain tissue at 400×, with Hematoxylin & Eosin staining, showing vacuolization of the neuropil, multiple parasitic structures in capillary lumens compatible with trophozoites.
